# Optimum non-invasive predictive indicators for metabolic dysfunction-associated fatty liver disease and its subgroups in the Chinese population: A retrospective case-control study

**DOI:** 10.3389/fendo.2022.1035418

**Published:** 2022-12-01

**Authors:** Jing Liu, Shaojie Duan, Che Wang, Yutong Wang, Hongye Peng, Zuohu Niu, Shukun Yao

**Affiliations:** ^1^ Graduate School, Peking Union Medical College, Beijing, China; ^2^ Department of Gastroenterology, China-Japan Friendship Hospital, Beijing, China; ^3^ Graduate School, Beijing University of Chinese Medicine, Beijing, China; ^4^ School of Qi Huang, Beijing University of Chinese Medicine, Beijing, China; ^5^ Department of Infection, Guang’anmen Hospital, China Academy of Chinese Medical Sciences, Beijing, China

**Keywords:** metabolic dysfunction-associated fatty liver disease, non-invasive predictive indicators, metabolic disorders, fatty liver index, lipid accumulation product, waist circumference-triglyceride index

## Abstract

**Objective:**

Metabolic dysfunction-associated fatty liver disease (MAFLD) affects 25% of the population without approved drug therapy. According to the latest consensus, MAFLD is divided into three subgroups based on different diagnostic modalities, including Obesity, Lean, and Type 2 diabetes mellitus (T_2_DM) MAFLD subgroups. This study aimed to find out the optimum non-invasive metabolism-related indicators to respectively predict MAFLD and its subgroups.

**Design:**

1058 Chinese participants were enrolled in this study. Anthropometric measurements, laboratory data, and ultrasonography features were collected. 22 metabolism-related indexes were calculated, including fatty liver index (FLI), lipid accumulation product (LAP), waist circumference-triglyceride index (WTI), etc. Logistic regression analyzed the correlation between indexes and MAFLD. Receiver operating characteristics were conducted to compare predictive values among 22 indicators for screening the best indicators to predict MAFLD in different subgroups.

**Results:**

FLI was the best predictor with the maximum odds ratio (OR) values of overall MAFLD (OR: 6.712, 95%CI: 4.766-9.452, area under the curve (AUC): 0.879, *P <* 0.05) and T_2_DM MAFLD subgroup (OR: 14.725, 95%CI: 3.712-58.420, AUC: 0.958, *P <* 0.05). LAP was the best predictor with the maximum OR value of Obesity MAFLD subgroup (OR: 2.689, 95%CI: 2.182-3.313, AUC: 0.796, *P <* 0.05). WTI was the best predictor with the maximum OR values of Lean MAFLD subgroup (OR: 3.512, 95%CI: 2.286-5.395, AUC: 0.920, *P <* 0.05).

**Conclusion:**

The best predictors of overall MAFLD, Obesity, Lean, and T_2_DM MAFLD subgroups were respectively FLI, LAP, WTI, and FLI.

## Introduction

Metabolic dysfunction-associated fatty liver disease (MAFLD) has become one of the most common chronic liver diseases and affected more than 25% of the population (1). The etiology of MAFLD is multifactorial and not understood completely, including genetic make-up, accumulation of intrahepatic lipids, insulin resistance (IR), and inflammatory responses (2). Furthermore, most patients with MAFLD do not have symptoms at an early stage, which limits the early detection and prevention of MAFLD. However, MAFLD is not a benign static liver disease. On one hand, it could progress silently to cirrhosis and liver-related death. On the other hand, as the hepatic manifestation of metabolic disorder, it affects the metabolic state of the body, which acts on the heart and cerebral vessels, increasing the risk of cardiovascular death (3).

Unfortunately, there are no approved therapies for MAFLD treatment (4). The early stages of MAFLD can be reversible with a healthy lifestyle. Hence, it is necessary to develop early identification, diagnosis, and lifestyle intervention. As the gold standard for diagnosis of MAFLD, the application of liver biopsy is limited because it is expensive and invasive with up to 1% risk of serious complications (5). Until now, abdominal ultrasound has been recommended as the preferred diagnostic method for fatty liver disease (FLD), which is still inconvenient for large populations (6).

Some anthropometric measures and lipid parameters are proven to be simple and useful tools for predicting metabolic syndrome (MS) in clinical practice (7). Previous studies suggested some indicators could predict hepatic steatosis, such as fatty liver index (FLI), etc. Nima Motamed et al. found that the FLI, as a noninvasive and relatively inexpensive index, had an acceptable ability to predict the occurrence of new cases of NAFLD in the study population from northern Iran (8). Besides, atherogenic index of plasma (AIP) was considered as a regular monitoring index of NAFLD for obese men due to its strong correlation with NAFLD in obese participants (9). However, most researchers only aimed at one or two parameters without comparison among more indexes (10, 11). The latest consensus suggests that there are three subgroups in the diagnosis of MAFLD, including Obesity, Lean, and T_2_DM MAFLD subgroups (12). Given the high complexity of the pathogenesis of MAFLD, we need specific diagnostic markers for MAFLD and its different subgroups, but they have been unclear yet. Hence, in this study, we compared the predictive values of 22 metabolic indicators and screened out specific indexes to predict MAFLD, especially discriminating different subgroups.

## Materials and methods

### Study population

This is a retrospective case-control study of 1,058 Chinese participants with or without MAFLD who were recruited consecutively at the China-Japan Friendship Hospital from January 2021 to February 2022. There were 621 patients with MAFLD and 437 participants with non-MAFLD. MAFLD and non-MAFLD control groups were respectively divided into Obesity, Lean and T_2_DM subgroups. In the MAFLD population, there were 464, 36, and 121 patients in the Obesity, Lean, and T_2_DM subgroups. In the non-MAFLD group, there were 244, 152, and 41 participants in the Obesity, Lean, and T_2_DM non-MAFLD subgroups.

### Study objects of MAFLD

Inclusion criteria were as follows: 1) Age ≥18 years and ≤80 years; 2) Diagnosis of MAFLD. The standards of diagnosis were as follows:

(1) Diagnosed with hepatic steatosis by ultrasound (13). At least two of the three conditions: ① hypoechogenicity in the far field of the liver; ② hyperechogenicity in the near field of the liver or bright liver, as well as signs of it being stronger than the kidney cortex; and ③ a blurry intrahepatic tubular structure.

(2) At least two of the three conditions: ① Obesity: BMI ≥23 kg/m^2^; ② Lean: BMI<23 kg/m^2^ and with at least two of the following metabolic risk abnormalities: a. Waist circumference ≥90/80 cm in Asian men and women; b. Blood pressure ≥130/85 mmHg or specific drug treatment; c. Plasma triglycerides ≥150 mg/dL (≥1.70 mmol/L) or specific drug treatment; d. Plasma HDL-C<40 mg/dL (<1.0 mmol/L) for men and<50 mg/dL (<1.3 mmol/L) for women or specific drug treatment; e. Prediabetes (i.e., fasting glucose levels 100-125 mg/dL (5.6-6.9 mmol/L), or 2-hour post-load glucose levels 140-199 mg/dL (7.8-11.0 mmol/L) or HbA1c 5.7%-6.4% (39-47 mmol/L); f. Homeostasis model assessment of insulin resistance score ≥2.5; g. Plasma high-sensitivity C-reactive protein level >2 mg/L. ③ T_2_DM: diagnosed with type 2 diabetes mellitus according to widely accepted international criteria (14).

Exclusion criteria: 1) Missing important information (such as age, gender or ultrasonography). 2) Cushing’s syndrome, total parenteral nutrition, drugs (amiodarone, ammonium valproate, glucocorticoids, methotrexate), etc., which can lead to special conditions of fatty liver. 3) Suffering from serious cardiovascular and cerebrovascular diseases, lung diseases, kidney diseases and so on. 4) Malignant tumors of the liver and other systems. 5) Pregnancy and lactation.

**Figure 1:**
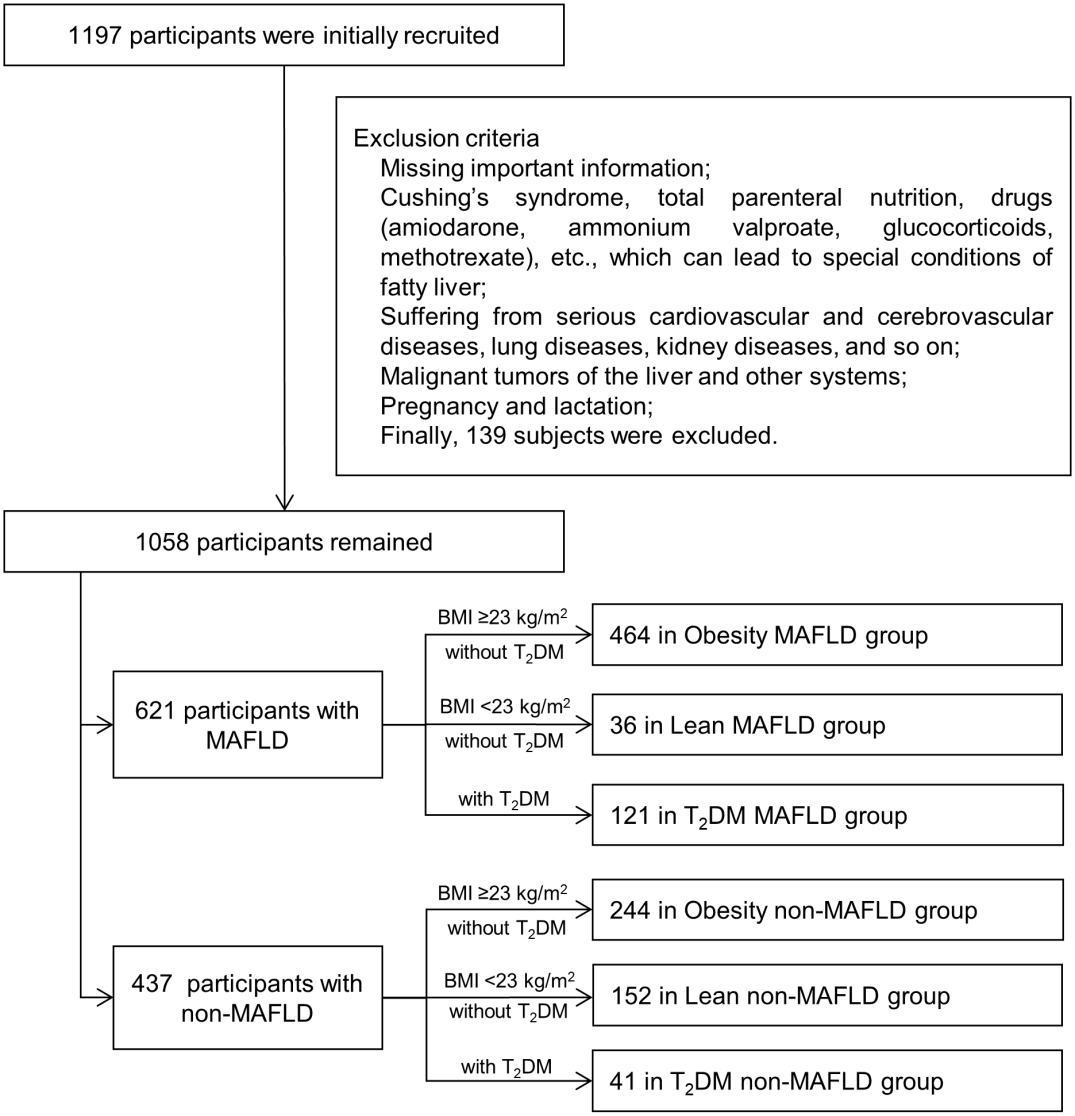
Flow diagram of research procedure. MAFLD, metabolic dysfunction-associated fatty liver disease; T_2_DM, type 2 diabetes mellitus.

The flow chart was shown in **Figure 1**.

### Definition of non-MAFLD controls

The definition of non-MAFLD controls was the individuals without fatty liver diagnosed by ultrasound. Obesity non-MAFLD control included obese individuals (BMI ≥23 kg/m^2^) without T_2_DM and fatty liver diagnosed by ultrasound. Lean non-MAFLD control included lean individuals (BMI <23 kg/m^2^) without T_2_DM and didn’t conform to the diagnostic criteria of MAFLD. T_2_DM non-MAFLD control included T_2_DM patients without fatty liver diagnosed by ultrasound.

### Ethics approval statement

The study was approved by China-Japan Friendship Hospital Clinical Research Ethics Committee (ID: 2018-110-K79-1).

### Data collection and measurements

The researchers administered a structured questionnaire to document specified data on demographic, health-related behaviors, previous history, and medication history. Anthropometric indices were measured by an eligible physician, including weight, height, waist circumference (WC), hip circumference (HC), and blood pressure (BP). Weight and height were measured in light indoor clothing without shoes and heavy clothes, using a calibrated measuring apparatus. WC was measured using an inelastic measuring tape at midline between the lowest rib and the iliac crest. HC was measured at the maximum extension of the buttocks. The BP was measured using automatic electronic sphygmomanometer with the arm supported at the level of the heart. The mean readings of three replicate measurements were recorded.

Health examinations were performed in the morning after the examinees fasted overnight. Laboratory evaluation included aspartate aminotransferase (AST), Alanine transaminase (ALT), Gamma-glutamyl transferase (GGT), low-density lipoprotein cholesterol (LDL-C), high-density lipoprotein cholesterol (HDL-C), total cholesterol (TC), triglyceride (TG), total bilirubin (TBil), direct bilirubin (DBil), blood glucose (GLU). We performed the blood tests once and obtained all the data. There was no interval.

### Definition of indicators

The calculation methods of the indicators were shown in **Table 1**.

**Table 1:** Definitions and calculation methods of indicators.

Indicators	Full name	Calculation methods
BMI	Body mass index	Wt/Ht^2^
AI1	Atherosclerosis index1	(TC−HDL-C)/HDL-C
AI2	Atherosclerosis index2	LDL-C/HDL-C
non-HDL-C	Non-high-density lipoprotein cholesterol	TC−HDL-C
R-CHR	Coronary heart index	TC/HDL-C
AIP	Atherogenic index of plasma	lg(TG/HDL-C)
LCI	Lipid comprehensive index	TC×TG×LDL-C/HDL-C
BLCI1	Bilirubin lipid composite index1	TC/(HDL-C+TBIL)
BLCI2	Bilirubin lipid composite index2	LDL-C/(HDL-C+TBIL)
TG/HDL-C	Triglyceride/(high-density lipoprotein cholesterol)	TG/HDL-C
BAI	Body adiposity index	HC/Ht^1.5^−18
BRI	Body roundness index	364.2−365.5×{1−[WC/(π×Ht)]^2^}^0.5^
LAP	Lipid accumulation product	(WC−65)×TG for males (WC−58)×TG for females
VAI	Visceral adiposity index	WC/(39.68 + 1.88×BMI)×(TG/1.03)×(1.31/HDL-C) for males WC/(36.58 + 1.89×BMI)×(TG/0.81)×(1.52/HDL-C) for females
WTI	Waist circumference-triglyceride index	WC×TG
WWI	Weight-adjusted waist index	WC/Wt^0.5^
CVAI	Chinese visceral adiposity index	−267.93+0.68×age+0.03×BMI+4.00×WC+22.00×Lg(TG)−16.32×HDL-C for males −187.32+1.71×age+4.23×BMI+1.12×WC+39.76×Lg(TG)−11.66×HDL-C for females
FLI	Fatty liver index	100/(1+e^-z^) z=0.953×lnTG+0.139×BMI+0.718×lnGGT+0.053×WC−15.745
ZJU index	Zhejiang University index	BMI+FPG+TG+3×ALT/AST for males BMI+FPG+TG+3×ALT/AST+2 for females
TyG index	Triglyceride-glucose index	ln(TG×FPG/2)
FLDI	Fatty liver disease index	BMI+TG+3×(ALT/AST)+2×hyperglycemia(HG) (presence of HG, HG=1; absence of HG, HG=0)
CMI	Cardiometabolic index	(WC/Ht)×(TG/HDL-C)

Wt, weight; Ht, height; TC, total cholesterol; HDL-C, high-density lipoprotein cholesterol; TG, triglyceride; LDL-C, low-density lipoprotein cholesterol; TBil, total bilirubin; HC, hip circumference; WC, waist circumference; GGT, gamma-glutamyl transferase; FPG, fasting plasma glucose; ALT, alanine transaminase; AST, aspartate aminotransferase.

### Statistical analyses

Data analysis was conducted using SPSS 26.0 and Medcalc 20.022 statistical software. Measurement data were expressed as mean ± standard deviation ( $\bar{x}\pm s$ ) and analyzed by student’s *t*-test. Counting data expressed as a percentage (%) were analyzed by chi-square test. *P<*0.05 was considered statistically significant.

Logistic regression was used to assess the relationship between indexes (including quartiles) and MAFLD, expressed by odds ratios (OR) with their 95% confidence intervals (CI). The values of OR were normalized. *Cramer’s* V was used to evaluate the strength of the relationship. It means a weak association when 0.1< *Cramer’s* V< 0.3, a medium association when 0.3 ≤ *Cramer’s* V< 0.5, a strong association when *Cramer’s* V ≥ 0.5. The area under the curves (AUCs) with a 95% CI of receiver operating characteristic (ROC) were calculated to compare the predictive value among indicators for screening the best indicators to predict MAFLD in different subgroups and determine optimal cutoff points and Youden index with maximum concomitant sensitivity and specificity. The case/control quartile trends of indexes were analyzed, and the indexes levels were stratified into 4 quartiles (quartile 1: ≤1st quartile; quartile 2: >1st quartile and ≤median; quartile 3: >median and ≤3rd quartile, and quartile 4: >3rd quartile). The lowest quartile (quartile 1) was defined as the reference category and compared with quartile 2, 3, and 4.

### Control methods of bias

In the design phase, we selected on-set FLD patients as cases as possible. When data were collected, repeated measurements were applied. In the analysis phase, a multivariate logistic regression analysis was used to control confounding factors such as age and gender. And the outcome assessors were blinded to the exposure status of participants and the study aims.

## Results

### The comparison of clinical parameters and composite indicators in groups

A total of 1058 subjects were included in this study, including 621 (58.69%) MAFLD patients and 437 (41.31%) non-MAFLD patients. Compared with non-MAFLD, MAFLD patients had higher TG, Glu, GGT, WC, HC, ALT, AST, and BP (**Table 2**), and the values of composite indexes, including BMI, AI2, AIP, LCI, BLCI2, TG/HDL-C, BAI, BRI, LAP, VAI, WTI, WWI, CVAI, FLI, ZJU index, TyG index, FLDI, and CMI (**Table 3**). In the Obesity, Lean, and T_2_DM subgroups, MAFLD patients had higher values of most of the composite indexes than respective non-MAFLD control (*P* all<0.05).

**Table 2:** Baseline characteristics of participants.

Indicators	MAFLD (n =621)	Non-MAFLD (n =437)	*P*
Age (years)	41.14 ± 10.93	39.74 ± 12.27	0.052
Weight (kg)	86.91 ± 17.13	70.76 ± 12.51	0.000
Height (m)	1.72 ± 0.08	1.71 ± 0.09	0.007
Male [n (%)]	462 (74.40)	285 (65.22)	0.001
Obesity MAFLD [n (%)]	464 (74.72)	244 (55.84)	0.000
Lean MAFLD [n (%)]	36 (5.80)	152 (34.78)	0.000
T_2_DM MAFLD [n (%)]	121 (19.48)	41 (9.38)	0.000
AST (U/L)	28.03 ± 16.78	20.81 ± 9.33	0.000
ALT (U/L)	46.77 ± 35.70	24.74 ± 16.35	0.000
GGT (U/L)	47.94 ± 35.00	32.22 ± 31.51	0.000
LDL-C (mmol/L)	2.90 ± 0.75	2.62 ± 0.73	0.000
HDL-C (mmol/L)	1.21 ± 0.49	1.35 ± 0.29	0.000
TC (mmol/L)	4.88 ± 0.99	4.57 ± 0.89	0.000
TG (mmol/L)	2.42 ± 2.07	1.24 ± 0.84	0.000
TBil (μmol/L)	13.73 ± 5.77	13.98 ± 6.42	0.511
DBil (μmol/L)	2.37 ± 1.03	2.30 ± 1.03	0.242
GLU (mmol/L)	6.33 ± 2.24	5.41 ± 1.15	0.000
WC (cm)	100.44 ± 11.85	85.67 ± 9.97	0.000
HC (cm)	111.02 ± 12.87	98.55 ± 7.80	0.000
SBP (mm Hg)	131.61 ± 19.48	125.69 ± 15.52	0.000
DBP (mm Hg)	83.08 ± 12.21	76.89 ± 11.74	0.000

Measurement data were expressed as mean ± standard deviation ( $\bar{x}\pm s$ ) and analyzed by student’s t-test. Counting data were expressed as number (percentage) and analyzed by chi-square test.

MAFLD, metabolic dysfunction-associated fatty liver disease; T_2_DM, type 2 diabetes mellitus; AST, aspartate aminotransferase; ALT, alanine transaminase; GGT, gamma-glutamyl transferase; LDL-C, low-density lipoprotein cholesterol; HDL-C, high-density lipoprotein cholesterol; TC, total cholesterol; TG, triglyceride; TBil, total bilirubin; DBil, direct bilirubin; GLU, glucose; WC, waist circumference; HC, hip circumference; SBP, systolic blood pressure; DBP, diastolic blood pressure.

**Table 3:** The calculation values of indexes for MAFLD and its subgroups.

	Overall (n =1058)		Obesity (n =708)		Lean (n =188)		T_2_DM (n =162)	
Indicators	MAFLD (n =621)	Non-MAFLD (n =437)	MAFLD (n =464)	Non-MAFLD (n =244)	MAFLD (n =36)	Non-MAFLD (n =152)	MAFLD (n =121)	Non-MAFLD (n =41)
BMI	29.29 ± 4.87	24.20 ± 3.09^*^	28.47 ± 2.90	26.13 ± 2.19^*^	22.68 ± 0.35	21.04 ± 1.42^*^	34.39 ± 6.93	24.41 ± 2.66^*^
AI1	3.25 ± 0.99	2.49 ± 0.88^*^	3.20 ± 0.95	2.67 ± 0.89^*^	2.97 ± 1.08	2.17 ± 0.78^*^	3.53 ± 1.06	2.62 ± 0.91^*^
AI2	2.56 ± 0.80	2.03 ± 0.72^*^	2.56 ± 0.78	2.20 ± 0.74^*^	2.43 ± 0.98	1.79 ± 0.64^*^	2.61 ± 0.84	1.91 ± 0.68^*^
non-HDL-C	3.67 ± 0.86	3.21 ± 0.85^*^	3.63 ± 0.84	3.27 ± 0.81^*^	3.67 ± 1.08	3.06 ± 0.87^*^	3.82 ± 0.86	3.42 ± 1.00^*^
R-CHR	4.25 ± 0.99	3.49 ± 0.88^*^	4.20 ± 0.95	3.67 ± 0.89^*^	3.97 ± 1.08	3.17 ± 0.78^*^	4.53 ± 1.06	3.62 ± 0.91^*^
AIP	0.24 ± 0.26	-0.09 ± 0.28^*^	0.21 ± 0.26	-0.02 ± 0.27^*^	0.18 ± 0.24	-0.22 ± 0.24^*^	0.35 ± 0.23	-0.06 ± 0.30^*^
LCI	30.75 ± 23.67	13.27 ± 13.18^*^	29.03 ± 23.17	15.33 ± 13.42^*^	27.87 ± 22.21	9.34 ± 8.99^*^	38.20 ± 24.68	15.59 ± 20.12^*^
BLCI1	0.37 ± 0.15	0.34 ± 0.14^*^	0.37 ± 0.15	0.34 ± 0.14^*^	0.44 ± 0.17	0.34 ± 0.14^*^	0.36 ± 0.16	0.33 ± 0.13
BLCI2	0.22 ± 0.10	0.20 ± 0.09^*^	0.22 ± 0.10	0.20 ± 0.09^*^	0.26 ± 0.12	0.19 ± 0.09^*^	0.21 ± 0.10	0.17 ± 0.08
TG/HDL-C	2.06 ± 1.25	1.00 ± 0.78^*^	1.94 ± 1.21	1.16 ± 0.85^*^	1.79 ± 1.13	0.71 ± 0.44^*^	2.58 ± 1.34	1.14 ± 1.00^*^
BAI	31.38 ± 6.54	26.38 ± 4.21^*^	30.94 ± 6.32	26.89 ± 4.11^*^	28.79 ± 7.55	25.59 ± 4.52^*^	33.87 ± 6.43	26.20 ± 3.04^*^
BRI	5.18 ± 1.67	3.46 ± 1.01^*^	4.80 ± 1.10	3.90 ± 0.87^*^	4.04 ± 1.22	2.67 ± 0.78^*^	6.98 ± 2.25	3.76 ± 0.72^*^
LAP	92.37 ± 90.87	30.56 ± 30.64^*^	76.42 ± 59.25	38.75 ± 35.26^*^	66.15 ± 53.04	16.32 ± 12.42^*^	161.34 ± 149.37	34.62 ± 30.01^*^
VAI	3.07 ± 1.90	1.44 ± 1.05^*^	2.80 ± 1.72	1.63 ± 1.14^*^	3.23 ± 2.17	1.10 ± 0.67^*^	4.08 ± 2.12	1.57 ± 1.29^*^
WTI	246.43 ± 220.71	108.78 ± 84.09^*^	217.66 ± 160.22	126.80 ± 95.40^*^	203.44 ± 157.54	75.87 ± 40.21^*^	369.54 ± 355.49	123.54 ± 96.70^*^
WWI	10.82 ± 0.80	10.22 ± 0.73^*^	10.68 ± 0.71	10.29 ± 0.65^*^	11.18 ± 1.18	10.02 ± 0.84^*^	11.28 ± 0.78	10.53 ± 0.46^*^
CVAI	142.55 ± 47.80	78.04 ± 45.61^*^	134.80 ± 35.49	96.84 ± 38.44^*^	95.28 ± 23.38	39.54 ± 33.33^*^	186.32 ± 62.85	108.93 ± 27.03^*^
FLI	10.05 ± 17.11	1.16 ± 2.36^*^	5.99 ± 7.86	1.75 ± 2.93^*^	1.11 ± 0.93	0.20 ± 0.25^*^	28.24 ± 29.20	1.17 ± 1.64^*^
ZJU index	43.35 ± 7.10	35.01 ± 4.20^*^	41.62 ± 4.21	37.10 ± 3.22^*^	35.69 ± 1.98	31.04 ± 2.17^*^	52.26 ± 9.05	37.24 ± 4.45^*^
TyG index	1.80 ± 0.70	1.04 ± 0.58^*^	1.66 ± 0.59	1.13 ± 0.55^*^	1.67 ± 0.51	0.79 ± 0.48^*^	2.40 ± 0.83	1.44 ± 0.70^*^
FLDI	37.14 ± 6.36	29.12 ± 4.30^*^	35.94 ± 4.20	31.47 ± 3.28^*^	29.35 ± 1.94	24.95 ± 2.25^*^	44.08 ± 8.22	30.59 ± 3.84^*^
CMI	1.21 ± 0.79	0.52 ± 0.43^*^	1.11 ± 0.71	0.62 ± 0.48^*^	0.95 ± 0.60	0.33 ± 0.21^*^	1.70 ± 0.92	0.60 ± 0.55^*^

Data were expressed as mean ± standard deviation ( $\bar{x}\pm s$ ) and analyzed by student’s t-test for continuous variables.

compared with MAFLD in respective subgroups, ^*^P < 0.05.

MAFLD, metabolic dysfunction-associated fatty liver disease; T_2_DM, type 2 diabetes mellitus; BMI, body mass index; AI, atherosclerosis index; HDL-C, high-density lipoprotein cholesterol; R-CHR, coronary heart index; AIP, atherogenic index of plasma; LCI: lipid comprehensive index; BLCI, bilirubin lipid composite index; TG, triglyceride; BAI, body adiposity index; BRI, body roundness index; LAP, lipid accumulation product; VAI, visceral fat index; WTI, waist triglyceride index; WWI, weight-adjusted waist index; CVAI, Chinese visceral adiposity index; FLI, fatty liver index; ZJU index, Zhejiang University index; TyG index, triglyceride-glucose index; FLDI, fatty liver disease index; CMI, cardiometabolic index.

### FLI was the best predictor of overall MAFLD

**Figure 2:**
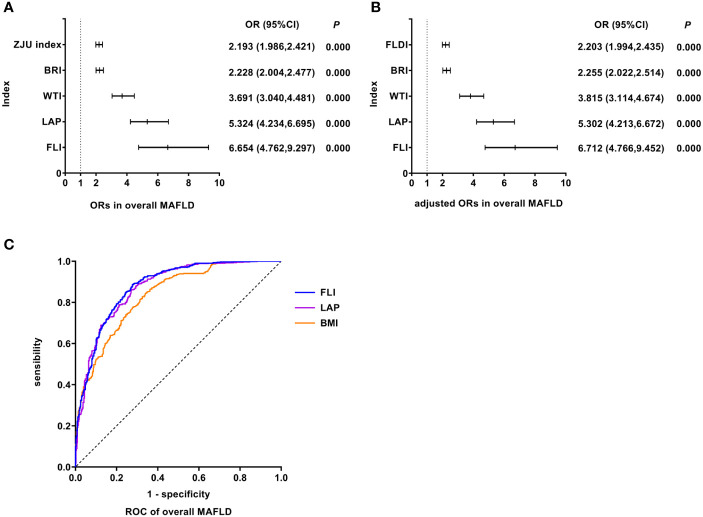
Logistic regression and ROC analysis of indicators in overall MAFLD. **(A)** ORs in overall MAFLD. **(B)** adjusted ORs in overall MAFLD. **(C)** ROC of overall MAFLD. MAFLD, metabolic dysfunction-associated fatty liver disease; OR, odds ratio; adjusted ORs: the values of ORs adjusted for gender and age (for age of LAP); ROC, receiver operating characteristic; ZJU index, Zhejiang University index; BRI, body roundness index; WTI, waist triglyceride index; LAP, lipid accumulation product; FLI, fatty liver index; FLDI, fatty liver disease index; BMI, body mass index.

As shown in **Figure 2**, univariate logistic regression results indicated that FLI had the strongest association with the overall MAFLD (unadjusted OR: 6.654, 95%CI: 4.762-9.297, *P<*0.05). After adjusting for age and gender, multivariate logistic regression analyses indicated that FLI was still the indicator with the strongest association (adjusted OR: 6.712, 95%CI: 4.766-9.452, *P<*0.05). ROC analyses found that the ability to predict substantially increased fatty liver burden was the strongest for FLI with the highest AUC value (AUC: 0.879, *P<*0.05). The ROC curves were plotted (see **Figure 2**), and the values of cutoff points, sensitivity, specificity, and Youden index of FLI were determined (see **Table 4**). In analyses of quartiles, as shown in **Table 5**, there were statistically significant differences in the prevalence of MAFLD in different FLI quartile groups. And the FLI level was strongly correlated to MAFLD, (*Cramer’s* V: 0.642). Taking the F1 group as a reference, after adjusting for gender and age, the risks of MAFLD in the F2, F3, and F4 groups were higher than that in the F1 group (*P* all<0.05). These results suggested that FLI was the best predictor of overall MAFLD and the risk of overall MAFLD enhanced with the increasing level of FLI.

**Table 4:** ROC analysis of indices in predicting the risk of MAFLD in different groups.

	Indexes	AUC	95%CI	Sensitivity(%)	Specificity(%)	Youden index	Cutoff points
MAFLD							
	FLI	0.879^*^	(0.857,0.898)	88.57	72.31	0.6088	0.915
	LAP	0.873^*^	(0.851,0.892)	86.15	72.54	0.5869	56.505
	BMI	0.836	(0.812,0.858)	84.38	65.90	0.5028	25.391
Obesity MAFLD							
	ZJU index	0.816^*^	(0.786,0.844)	71.12	80.33	0.5145	39.139
	LAP	0.796^*^	(0.765,0.826)	64.01	82.79	0.4680	50.700
	BMI	0.748	(0.715,0.780)	62.50	72.54	0.3504	27.143
Lean MAFLD							
	ZJU index	0.952^*^	(0.912,0.978)	88.89	89.47	0.7836	33.706
	WTI	0.920	(0.872,0.954)	80.56	89.47	0.7003	120.120
	BMI	0.890	(0.836,0.931)	86.11	83.55	0.6966	22.477
T_2_DM MAFLD							
	BRI	0.963	(0.921,0.986)	81.82	100.00	0.8182	4.970
	FLI	0.958	(0.915,0.983)	90.91	87.80	0.7871	2.071
	BMI	0.953	(0.909,0.980)	96.69	78.05	0.7474	25.992

The predictive abilities of indicators were analyzed by ROC.

compared with BMI in respective subgroups, ^*^P < 0.05.

MAFLD, metabolic dysfunction-associated fatty liver disease; T_2_DM, type 2 diabetes mellitus; ROC, receiver operating characteristic; AUC, areas under the curve; CI, confidence interval; FLI, fatty liver index; LAP, lipid accumulation product; BMI, body mass index; ZJU index, Zhejiang University index; WTI, waist triglyceride index; BRI, body roundness index.

**Table 5:** Prevalence of MAFLD and its subgroups in quartile groups of different indexes [n(%)].

	FLI				χ^2^	*P*	*Cramer’s* V
	F1 (n=265)	F2 (n=264)	F3 (n=264)	F4 (n=265)			
MAFLD	27 (10.19)	137 (51.89)*^a^*	213 (80.68)*^ab^*	244 (92.08)*^abc^*	436.655	<0.01	0.642
non-MAFLD	238 (89.81)	127 (48.11)*^a^*	51 (19.32)*^ab^*	21 (7.92)*^abc^*			
OR		9.509^*^	36.815^*^	102.420^*^			
adjusted OR		12.830^*^	49.884^*^	149.589^*^			
	LAP						
	L1 (n=265)	L2 (n=264)	L3 (n=264)	L4 (n=265)			
Obesity MAFLD	21 (7.92)	125 (47.35)*^a^*	165 (62.50)*^ab^*	153 (57.74)*^abc^*	175.858	<0.01	0.498
Obesity non-MAFLD	89 (33.58)	95 (35.98)*^a^*	44 (16.67)*^ab^*	16 (6.04)*^abc^*			
OR		5.576^*^	15.893^*^	40.527^*^			
adjusted OR		5.521^*^	15.461^*^	39.894^*^			
	WTI						
	W1 (n=265)	W2 (n=264)	W3 (n=264)	W4 (n=265)			
Lean MAFLD	3 (1.13)	8 (3.03)*^a^*	19 (7.20)*^ab^*	6 (2.26)*^ab^*	78.098	<0.01	0.645
Lean non-MAFLD	111 (41.89)	29 (10.98)*^a^*	11 (4.17)*^ab^*	1 (0.38)*^ab^*			
OR		10.207^*^	63.909^*^	222.000^*^			
adjusted OR		12.980^*^	82.272^*^	221.349^*^			
	FLI						
	F1 (n=265)	F2 (n=264)	F3 (n=264)	F4 (n=265)			
T_2_DM MAFLD	2 (0.75)	5 (1.89)	22 (8.33)*^ab^*	92 (34.72)*^abc^*	97.985	<0.01	0.778
T_2_DM non-MAFLD	20 (7.55)	13 (4.92)	6 (2.27)*^ab^*	2 (0.75)*^abc^*			
OR		3.846	36.667^*^	460.000^*^			
adjusted OR		4.962	70.418^*^	321.323^*^			

The correlations between the indexes and MAFLD groups in quartile groups were analyzed by univariate and multivariate logistic regression. Data were expressed as number (percentage) and analyzed by chi-square test for categorical variables.

compared with quartile 1 of predictors, ^*^P < 0.05; compared with quartile 1 of predictors, ^a^P < 0.05; compared with quartile 2 of predictors, ^b^P < 0.05; compared with quartile 3 of predictors, ^c^P <0.05.

adjusted OR: the values of ORs after adjusting for age and gender in FLI and WTI or age in LAP.

MAFLD, metabolic dysfunction-associated fatty liver disease; OR, odds ratio; T_2_DM, type 2 diabetes mellitus; FLI, fatty liver index; LAP, lipid accumulation product; WTI, waist circumference-triglyceride index.

### LAP was the best predictor of Obesity MAFLD

**Figure 3:**
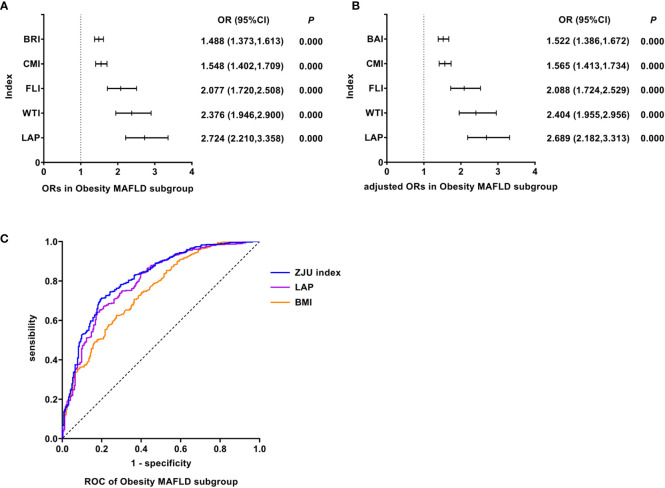
Logistic regression and ROC analysis of indicators in Obesity MAFLD subgroup. **(A)** ORs in Obesity MAFLD subgroup. **(B)** adjusted ORs in Obesity MAFLD subgroup. **(C)** ROC of Obesity MAFLD subgroup. MAFLD, metabolic dysfunction-associated fatty liver disease; OR, odds ratio; adjusted ORs: the values of ORs adjusted for gender and age (for age of LAP); ROC, receiver operating characteristic; BRI, body roundness index; CMI, cardiometabolic index; FLI, fatty liver index; WTI, waist triglyceride index; LAP, lipid accumulation product; BAI, body adiposity index; ZJU index, Zhejiang University index; LAP, lipid accumulation product; BMI, body mass index.

As shown in **Figure 3**, univariate logistic regression results indicated that LAP had the strongest association with the Obesity MAFLD (unadjusted OR: 2.724, 95%CI: 2.210-3.358, *P<*0.05). After adjusting for age, multivariate logistic regression analyses indicated that LAP still was the indicator with the strongest association (adjusted OR: 2.689, 95%CI: 2.182-3.313, *P<*0.05). In Obesity MAFLD, ROC analyses found that the AUC of LAP was smaller than that of ZJU index, but the difference was not statistically significant (AUC: LAP vs. ZJU index: 0.796 vs. 0.816, *P >*0.05). The ROC curves were plotted (see **Figure 3**), and the values of cutoff points, sensitivity, specificity and Youden index of LAP were determined (see **Table 4**). In analyses of quartiles, as shown in **Table 5**, there were statistically significant differences in the prevalence of Obesity MAFLD in different LAP quartile groups. And the LAP level was moderately correlated to Obesity MAFLD (*Cramer’s* V: 0.498). Taking the L1 group as a reference, after adjusting for age, the risks of Obesity MAFLD in the L2, L3, and L4 groups were higher than that in the L1 group (*P* all<0.05). These results suggested that LAP was the best predictor of Obesity MAFLD and the risk of Obesity MAFLD enhanced with the increasing level of LAP.

### WTI was the best predictor of Lean MAFLD

**Figure 4:**
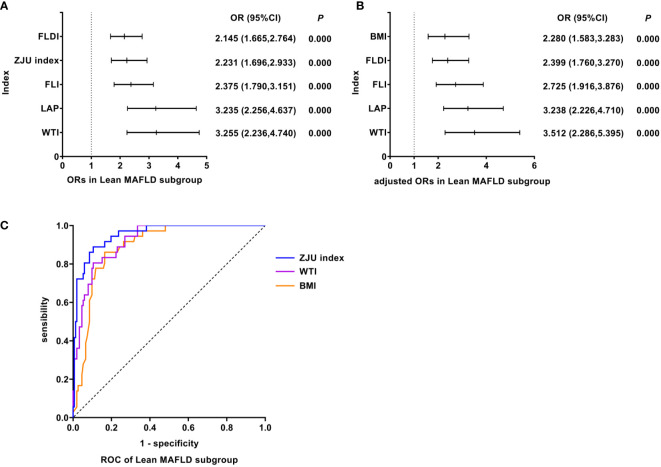
Logistic regression and ROC analysis of indicators in Lean MAFLD subgroup. **(A)** ORs in Lean MAFLD subgroup. **(B)** adjusted ORs in Lean MAFLD subgroup. **(C)** ROC of Lean MAFLD subgroup. MAFLD, metabolic dysfunction-associated fatty liver disease; OR, odds ratio; adjusted ORs: the value of ORs adjusted for gender and age (for age of LAP); ROC, receiver operating characteristic; FLDI, fatty liver disease index; ZJU index, Zhejiang University index; FLI, fatty liver index; LAP, lipid accumulation product; WTI, waist triglyceride index; BMI, body mass index; OR, odds ratio; adjusted ORs: The value of ORs after being adjusted for gender and age; ROC, receiver operating characteristic; MAFLD, metabolic dysfunction-associated fatty liver disease.

As shown in **Figure 4**, univariate logistic regression results indicated that WTI had the strongest association with the Lean MAFLD (unadjusted OR: 3.255, 95%CI: 2.236-4.740, *P<*0.05). After adjusting for age and gender, multivariate logistic regression analyses indicated that WTI still was the indicator with the strongest association (adjusted OR: 3.512, 95%CI: 2.286-5.395, *P<*0.05). In Lean MAFLD, ROC analyses found that the AUC of WTI was smaller than that of ZJU index, but the difference was not statistically significant (AUC: WTI vs. ZJU index: 0.920 vs. 0.952, *P >* 0.05). The ROC curves were plotted (see **Figure 4**), and the values of cutoff points, sensitivity, specificity, and Youden index of WTI were determined (see **Table 4**). In analyses of quartiles, as shown in **Table 5**, there were statistically significant differences in the prevalence of Lean MAFLD in different WTI quartile groups. And the WTI level was moderately correlated to Lean MAFLD (*Cramer’s* V: 0.645). Taking the W1 group as a reference, after adjusting for age and gender, the risks of Lean MAFLD in the W2, W3, W4 groups were higher than that in the W1 group (*P* all<0.05). These results suggested that WTI was the best predictor of Lean MAFLD and the risk of Lean MAFLD enhanced with the increasing level of WTI.

### FLI was the best predictor of T_2_DM MAFLD

**Figure 5:**
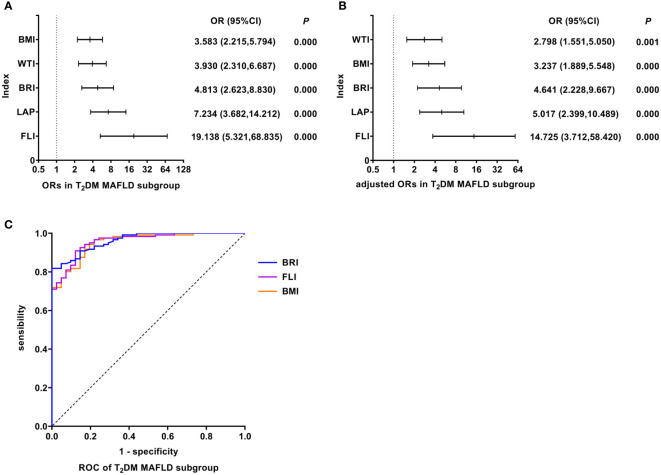
Logistic regression and ROC analysis of indicators in T_2_DM MAFLD subgroup. **(A)** ORs in T_2_DM MAFLD subgroup. **(B)** adjusted ORs in T_2_DM MAFLD subgroup. **(C)** ROC of T_2_DM MAFLD subgroup. MAFLD, metabolic dysfunction-associated fatty liver disease; T_2_DM, type 2 diabetes mellitus; OR, odds ratio; adjusted ORs: the value of ORs adjusted for gender and age (for age of LAP); ROC, receiver operating characteristic; BMI, body mass index; WTI, waist triglyceride index; BRI, body roundness index; LAP, lipid accumulation product; FLI, fatty liver index.

As shown in **Figure 5**, univariate logistic regression results indicated that FLI had the strongest association with the T_2_DM MAFLD (unadjusted OR: 19.138, 95%CI: 5.321-68.835, *P<*0.05). After adjusting for age and gender, multivariate logistic regression analyses indicated that FLI still was the indicator with the strongest association (adjusted OR: 14.725, 95%CI: 3.712-58.420, *P<*0.05). In T_2_DM MAFLD, ROC analyses found that the AUC of FLI was smaller than that of BRI, but the difference was not statistically significant (AUC: FLI vs. BRI: 0.958 vs. 0.963, *P >*0.05). The ROC curves were plotted (see **Figure 5**), and the values of cutoff points, sensitivity, specificity, and Youden index of FLI were determined (see **Table 4**). In analyses of quartiles, as shown in **Table 5**, there were statistically significant differences in the prevalence of T_2_DM MAFLD in different FLI quartile groups. And the FLI level was strongly correlated to T_2_DM MAFLD, (*Cramer’s* V: 0.778). Taking the F1 group as a reference, after adjusting for age and gender, the risks of T_2_DM MAFLD in the F3 and F4 groups were higher than that in the F1 group (*P* all< 0.05). These results suggested that FLI was the best predictor of T_2_DM MAFLD, and the risk of T_2_DM MAFLD enhanced with the increasing level of FLI.

The results of other indicators were shown in **Supplementary Material**.

## Discussion

It is the first step to recognizing fatty liver in screening people at high risk for steatohepatitis. Due to differences in the distribution of metabolic risk factors in population with discriminating characteristics, the performances of diagnostic models are also distinguished. We analyzed and compared multiple composite measurements to determine the best predictive tools for different subgroups of MAFLD.

### FLI was the best predictor of overall MAFLD and T_2_DM MAFLD

FLI is an algorithm combining BMI, WC, GGT, and TG levels, originally developed in a population residing in northern Italy and proposed by Bedogni to predict fatty liver (15). GGT is an early and sensitive marker of oxidative stress associated well with the levels of prooxidants, which can serve as an indirect marker of the risk of future oxidative damage (16). The high FLI level is related to increased C-reactive protein (CRP), blood pressure, IR, and fibrinogen levels (17).

Research conducted by Otgonsuren suggested FLI provided good diagnostic accuracy for fatty liver since it showed high concordance with the imaging and histological criteria for fatty liver (18). Kabisch et al. found that FLI could predict most cases of NAFLD with an AUC of 0.84 in ROC analysis (10). Nima’s study in northern Iran showed the ability of FLI to predict new cases of NAFLD was superior to other indexes such as BMI, WC, WHR, WHtR, and LAP. In their 7 years of follow-up, a one-unit increase in the FLI enhanced the chance of the occurrence of NAFLD by 3.8% in men and 3.2% in women (8). A meta-analysis of ten studies was conducted by Marco, evaluating the performance of FLI among 8273 subjects diagnosed with and 18,948 subjects without NAFLD, in which FLI showed an adequate performance in stratifying the risk of NAFLD (19).

Our study obtained supportive results that FLI was the best predictor of overall MAFLD and T_2_DM MAFLD, which suggested that the key mechanism of MAFLD may be strongly associated with IR. In a meta-analysis of 70,198 participants including 27 cohort studies, individuals with higher FLI levels had a significantly higher risk of diabetes development (20). Gerhard found patients with T_2_DM had a higher risk of progressive NAFLD with abnormal lipid metabolism and IR. Due to IR, adipose tissue becomes resistant to the anti-adipose effects of insulin to promote the synthesis of free fatty acids and TG in liver cells, which contributes to the accumulation of fat in liver cells, ultimately leading to FLD (21).

In America, the leading cause of mortality in patients with NAFLD was cardiovascular disease (22). FLD has been considered an early mediator of atherosclerosis (23, 24). Coexisting T_2_DM with FLD could aggravate liver diseases and atherosclerosis. Risks of cardiovascular events and kidney disease were enhanced in T_2_DM MAFLD patients (25). Lerchbaum et al. observed that a high level of FLI could predict all-cause, cardiovascular as well as hepatic mortality (17). FLI can confirm the diagnosis of MAFLD and evaluate the risks of related complications. Therefore, we contend that FLI is the most appropriate diagnostic index for overall MAFLD and T_2_DM MAFLD subgroup.

### LAP was the best predictor of obesity MAFLD

The most common explanation for the high prevalence of FLD is the obesity epidemic and weight loss is related to improvement in histology features of FLD (26). IR and excess adiposity increased lipid influx into the liver and *de novo* hepatic lipogenesis to promote hepatic triglyceride accumulation (27). It is generally recognized that serious complications of FLD (i.e., cirrhosis and liver cancer) are common in middle age and older. Whereas obesity and its related metabolic disorders could accelerate the process of disease (28). Obesity MAFLD will expose adolescents and young adults to the long-term risk of MAFLD, progressive hepatic lipotoxicity, and future end-stage liver disease (29, 30). Hence, it is significant to use the LAP index to screen MAFLD for obese people.

In a cross-sectional study involving 40,459 participants from southern China, LAP showed a strong association with the diagnosis and severity of NAFLD (31), in line with findings from northern China (11). Besides, LAP is one of the few indicators that can reflect sex differences in FLD, which is calculated for males and females respectively. Sex hormones and regional fat distribution take part in regulating metabolic disorders and FLD (32). In Liu’s study, LAP exhibited a stronger correlation with FLD in females than in males. The risk of FLD tended to increase in females who were about 55 years old (11). During this period, most women underwent menopause, during which estrogen levels sharply fell and body fat distribution shifted to the abdominal region leading to the increase of WC. Estrogen could regulate lipid metabolism and inhibit inflammation and plaque advancement in premenopausal women. Menopausal triggered a cascade of biological and physiological alterations, including fat redistribution (i.e., accumulation of visceral fat), dyslipidemia, and glucose intolerance, which are correlated with enhanced IR, cardiovascular disease, and NAFLD (32).

### WTI was the best predictor of Lean MAFLD

Lean MAFLD refers to FLD with normal BMI and metabolic disorders, involving WC, BP, TG, HDL-C, prediabetes, IR scores, and high-sensitivity CRP level. We found the ability to predict Lean MAFLD was strongest for WTI, which was based on WC and TG. As reported by Lee, WC and TG levels elevated significantly with the severity of hepatic steatosis. Furthermore, abdominal obesity with increased WC is a major manifestation of Lean MAFLD and appears to be associated with poorer metabolic status than general obesity and increased hepatic fibrosis (33). Excess VAT and SAT serve as two remarkable characteristics of abdominal obesity. Compared with SAT, the lipolytic activity is stronger in VAT, which plays a leading role in the development of Lean MAFLD (34). VAT induces the synthesis of cytokines like IL-6 and TNF-α, promoting macrophage infiltration and chronic inflammation (35). The signal transduction pathway of surrounding cells like T cells, eosinophils, B-regulatory cells, and macrophages was activated, leading to IR, liver steatosis, and eventually induced and deteriorated NAFLD (36). What’s more, Chinese individuals have a greater amount of VAT than Europeans at the same level of BMI or WC (37).

According to the NHANES III survey in the US, Kahn et al. found that people with high WC and TG had higher levels of fasting insulin and fasting plasma glucose than those with normal WC and TG. Excessive lipid accumulation reflected by WC and TG caused metabolic abnormalities (38). A Chinese study showed WTI was a better predictor of MetS in both men and women (39). In addition, WTI was also a predictor for the development of coronary artery disease (40). A mild to moderate increase in TG can reflect an increase in chylomicrons and very low density lipoprotein cholesterol remnants. Meanwhile, the particles of the remnant lipoprotein become smaller, they may directly cause atherosclerosis. Previous reports have noted that simultaneously increased WC and TG were associated with each metabolic index in CHD patients and the severity of lesions in the coronary artery (41, 42).

Individuals with Lean MAFLD may have worse outcomes, incorporating enhanced incidence of hepatitis, cardiometabolic complications, and even mortality, than those with metabolically healthy obese (43). Unfortunately, due to deceptive BMI and obscure metabolic abnormalities, patients with Lean MAFLD often go undetected and missed diagnosis at an early stage, to subsequently suffering from more severe diseases. Hence, as an easily available and the most accurate measurement of Lean MAFLD, WTI is demanding in the Chinese population, especially those whose BMI is normal.

### BMI was not a valuable diagnostic indicator for MAFLD overall

Previous studies compared the predictive ability of AIP, AVI, BAI, BMI, BRI, and TyG index, usually the ROAUC of BMI was the maximum (44). On the contrary, our study found that BMI was not in the top 3 for maximum OR in all the MAFLD groups. Additionally, the diagnosis ability of BMI was poorer than other indexes in all groups with lower AUCs. Although BMI used to be an anthropometric indicator of obesity and FLD, increasing evidence suggests that BMI is criticized for its single estimate of the degree of body adipose tissue, and Asians are more likely to have central fat deposition even with a lower BMI. It does neither reflect the status of metabolic disorders nor specifically distinguish among different subtypes of MAFLD. Hence, it is conceivable that BMI is not suitable for diagnosing metabolic disorders, such as MAFLD, especially in Asians.

### Strengths

MAFLD is considered as a hepatic manifestation of metabolic disorders. In this study, almost all commonly used anthropometric and metabolic indicators related to metabolic disorders were compared as far as possible. What’s more, we found specific diagnostic indicators for various subtypes of MAFLD, which were rarely seen in other studies, providing more accurate information and treatment measures for the performance of personalized protocols in clinical practice and public health consultation. In addition, simple alternative markers are significant for screening MAFLD at an early stage, while CT and MRI are not readily available due to the cost of money and time, especially in rural and grassroots areas of China with large population bases and relatively inadequate health resources. Our findings provide simple and inexpensive ways to identify and distinguish subtypes of MAFLD and monitor the risks of MAFLD and associated complications.

### Limitations

Firstly, we could not obtain results of the biopsy considered the gold standard of FLD, because in Chinese and other countries’ guidelines, liver biopsy is not recommended and applicable to all participants. As hepatic steatosis was evaluated using hepatic ultrasonography, the accuracy of the reference test might be reduced. Hence, the index test results can also be affected. Secondly, due to the observational study design, causality cannot be confirmed nor negated. Most included noninvasive indexes and scores used parameters that do not directly reflect the processes involved in hepatic steatosis and liver fibrosis, which might mean that these noninvasive indexes and scores do not directly indicate hepatic steatosis or liver fibrosis changes. In further studies, we would perform the prospective study to explore the proper noninvasive index to distinguish NASH or liver fibrosis and clarify the causality. Thirdly, the increased values of several hepatic steatosis scores and indexes that contain age in the formula, can partially be attributed to the increased age in different subgroups. And the severity of MAFLD was not classified, which prevented us from quantitatively evaluating steatosis. Besides, the data of other confounders including drinking status and exercise were not analyzed because relative information was not available. Finally, given that our results were predominantly applicable to Chinese adults, generalizability to other racial or ethnic populations is uncertain.

## Conclusion

The best predictors of overall MAFLD, Obesity, Lean, and T_2_DM MAFLD subgroups were respectively FLI, LAP, WTI, and FLI.

## Data availability statement

The original contributions presented in the study are included in the article/**Supplementary Material**. Further inquiries can be directed to the corresponding author.

## Ethics statement

The studies involving human participants were reviewed and approved by China-Japan Friendship Hospital Clinical Research Ethics Committee. The patients/participants provided their written informed consent to participate in this study.

## Author contributions

JL designed the study, analyzed the data, and wrote the manuscript. SD analyzed the data and revised the manuscript. CW, YW, HP, and ZN collected the data. SY designed the study and revised the manuscript. All authors read and approved the final manuscript.

## Funding

Science and Technology Project Task Book of Beijing, No. Z171100001717008.

## Acknowledgments

The authors thank the China-Japan Friendship Hospital for support.

## Conflict of interest

The authors declare that the research was conducted in the absence of any commercial or financial relationships that could be construed as a potential conflict of interest.

## Publisher’s note

All claims expressed in this article are solely those of the authors and do not necessarily represent those of their affiliated organizations, or those of the publisher, the editors and the reviewers. Any product that may be evaluated in this article, or claim that may be made by its manufacturer, is not guaranteed or endorsed by the publisher.

## Abbreviations

BMI, Body mass index; AI1, Atherosclerosis index1; AI2, Atherosclerosis index2; non-HDL-C, Non-high-density lipoprotein cholesterol; R-CHR, Coronary heart index; AIP, Atherogenic index of plasma; LCI, Lipid comprehensive index; BLCI1, Bilirubin lipid composite index1; BLCI2, Bilirubin lipid composite index2; TG/HDL-C, Triglyceride/(high-density lipoprotein cholesterol); BAI, Body adiposity index; BRI, Body roundness index; LAP, Lipid accumulation product; VAI, Visceral adiposity index; WTI, Waist circumference-triglyceride index; WWI, Weight-adjusted waist index; CVAI, Chinese visceral adiposity index; FLI, Fatty liver index; ZJU index, Zhejiang University index; TyG index, Triglyceride-glucose index; FLDI, Fatty liver disease index.
