# Supplementary material for: Optimum non-invasive predictive indicators for metabolic dysfunction-associated fatty liver disease and its subgroups in the Chinese population: A retrospective case-control study
Source: Front Endocrinol (Lausanne). 2022 Dec 1;13:1035418. doi: 10.3389/fendo.2022.1035418 (PMC9751395; doi:10.3389/fendo.2022.1035418)
Supplement: Supplementary file 1 [file DataSheet_1.docx]

Supplemental Materials

**The file includes:**

Table S1. Logistic regression analysis of assessing the relationship between 22 indices and MAFLD

Table S2. Logistic regression analysis of assessing the relationship between 22 indices and MAFLD with adjusted age and gender

Table S3. Prevalence of MAFLD and its subgroups in FLI quartile groups [n(%)]

Table S4. Prevalence of MAFLD and its subgroups in LAP quartile groups [n(%)]

Table S5. Prevalence of MAFLD and its subgroups in WTI quartile groups [n(%)]

Table S6. ROC analysis of 22 indices in predicting the risk of MAFLD and its subgroups

**Table S1. Logistic regression analysis of assessing the relationship between 22 indices and MAFLD**

|  | Indices | OR | 95%CI | P |
| --- | --- | --- | --- | --- |
| MAFLD |  |  |  |  |
|  | BMI | 2.156 | (1.949,2.386) | <0.05 |
|  | AI1 | 1.264 | (1.213,1.317) | <0.05 |
|  | AI2 | 1.220 | (1.173,1.269) | <0.05 |
|  | non-HDL-C | 1.190 | (1.141,1.242) | <0.05 |
|  | R-CHR | 1.264 | (1.213,1.317) | <0.05 |
|  | AIP | 1.353 | (1.300,1.408) | <0.05 |
|  | LCI | 1.558 | (1.446,1.678) | <0.05 |
|  | BLCI1 | 1.076 | (1.033,1.121) | <0.05 |
|  | BLCI2 | 1.100 | (1.054,1.148) | <0.05 |
|  | TG/HDL-C | 1.614 | (1.498,1.739) | <0.05 |
|  | BAI | 1.444 | (1.358,1.536) | <0.05 |
|  | BRI | 2.228 | (2.004,2.477) | <0.05 |
|  | LAP | 5.324 | (4.234,6.695) | <0.05 |
|  | VAI | 1.722 | (1.588,1.867) | <0.05 |
|  | WTI | 3.691 | (3.040,4.481) | <0.05 |
|  | WWI | 1.477 | (1.378,1.583) | <0.05 |
|  | CVAI | 1.800 | (1.670,1.941) | <0.05 |
|  | FLI | 6.654 | (4.762,9.297) | <0.05 |
|  | ZJU index | 2.193 | (1.986,2.421) | <0.05 |
|  | TyG index | 1.555 | (1.464,1.653) | <0.05 |
|  | FLDI | 2.071 | (1.891,2.269) | <0.05 |
|  | CMI | 1.863 | (1.701,2.040) | <0.05 |
| Obesity  MAFLD |  |  |  |  |
|  | BMI | 1.342 | (1.263,1.426) | <0.05 |
|  | AI1 | 1.185 | (1.128,1.246) | <0.05 |
|  | AI2 | 1.145 | (1.093,1.200) | <0.05 |
|  | non-HDL-C | 1.159 | (1.097,1.225) | <0.05 |
|  | R-CHR | 1.185 | (1.128,1.246) | <0.05 |
|  | AIP | 1.245 | (1.191,1.302) | <0.05 |
|  | LCI | 1.401 | (1.286,1.525) | <0.05 |
|  | BLCI1 | 1.061 | (1.008,1.117) | <0.05 |
|  | BLCI2 | 1.074 | (1.019,1.132) | <0.05 |
|  | TG/HDL-C | 1.400 | (1.291,1.519) | <0.05 |
|  | BAI | 1.351 | (1.255,1.454) | <0.05 |
|  | BRI | 1.488 | (1.373,1.613) | <0.05 |
|  | LAP | 2.724 | (2.210,3.358) | <0.05 |
|  | VAI | 1.463 | (1.340,1.596) | <0.05 |
|  | WTI | 2.376 | (1.946,2.900) | <0.05 |
|  | WWI | 1.339 | (1.229,1.459) | <0.05 |
|  | CVAI | 1.366 | (1.289,1.447) | <0.05 |
|  | FLI | 2.077 | (1.720,2.508) | <0.05 |
|  | ZJU index | 1.462 | (1.370,1.560) | <0.05 |
|  | TyG index | 1.343 | (1.266,1.425) | <0.05 |
|  | FLDI | 1.448 | (1.358,1.543) | <0.05 |
|  | CMI | 1.548 | (1.402,1.709) | <0.05 |
| Lean MAFLD |  |  |  |  |
|  | BMI | 1.981 | (1.477,2.658) | <0.05 |
|  | AI1 | 1.219 | (1.114,1.334) | <0.05 |
|  | AI2 | 1.205 | (1.102,1.317) | <0.05 |
|  | non-HDL-C | 1.131 | (1.053,1.215) | <0.05 |
|  | R-CHR | 1.219 | (1.114,1.334) | <0.05 |
|  | AIP | 1.478 | (1.300,1.681) | <0.05 |
|  | LCI | 1.339 | (1.193,1.503) | <0.05 |
|  | BLCI1 | 1.140 | (1.058,1.228) | <0.05 |
|  | BLCI2 | 1.151 | (1.072,1.236) | <0.05 |
|  | TG/HDL-C | 1.738 | (1.440,2.098) | <0.05 |
|  | BAI | 1.133 | (1.042,1.233) | <0.05 |
|  | BRI | 1.709 | (1.420,2.057) | <0.05 |
|  | LAP | 3.235 | (2.256,4.637) | <0.05 |
|  | VAI | 1.882 | (1.515,2.337) | <0.05 |
|  | WTI | 3.255 | (2.236,4.740) | <0.05 |
|  | WWI | 1.467 | (1.265,1.703) | <0.05 |
|  | CVAI | 1.536 | (1.333,1.769) | <0.05 |
|  | FLI | 2.375 | (1.790,3.151) | <0.05 |
|  | ZJU index | 2.231 | (1.696,2.933) | <0.05 |
|  | TyG index | 1.787 | (1.475,2.166) | <0.05 |
|  | FLDI | 2.145 | (1.665,2.764) | <0.05 |
|  | CMI | 1.820 | (1.493,2.219) | <0.05 |
| T_2_DM MAFLD |  |  |  |  |
|  | BMI | 3.583 | (2.215,5.794) | <0.05 |
|  | AI1 | 1.224 | (1.116,1.343) | <0.05 |
|  | AI2 | 1.206 | (1.106,1.316) | <0.05 |
|  | non-HDL-C | 1.097 | (1.017,1.183) | <0.05 |
|  | R-CHR | 1.224 | (1.116,1.343) | <0.05 |
|  | AIP | 1.366 | (1.233,1.514) | <0.05 |
|  | LCI | 1.361 | (1.188,1.560) | <0.05 |
|  | BLCI1 | 1.056 | (0.976,1.141) | >0.05 |
|  | BLCI2 | 1.078 | (0.996,1.167) | >0.05 |
|  | TG/HDL-C | 1.429 | (1.238,1.648) | <0.05 |
|  | BAI | 1.883 | (1.487,2.385) | <0.05 |
|  | BRI | 4.813 | (2.623,8.830) | <0.05 |
|  | LAP | 7.234 | (3.682,14.212) | <0.05 |
|  | VAI | 1.905 | (1.510,2.403) | <0.05 |
|  | WTI | 3.930 | (2.310,6.687) | <0.05 |
|  | WWI | 1.453 | (1.246,1.695) | <0.05 |
|  | CVAI | 1.753 | (1.446,2.124) | <0.05 |
|  | FLI | 19.138 | (5.321,68.835) | <0.05 |
|  | ZJU index | 2.092 | (1.624,2.695) | <0.05 |
|  | TyG index | 1.407 | (1.238,1.598) | <0.05 |
|  | FLDI | 2.382 | (1.759,3.226) | <0.05 |
|  | CMI | 1.646 | (1.375,1.971) | <0.05 |

The correlations between the indexes and MAFLD groups were analyzed by univariate logistic regression.

MAFLD: metabolic dysfunction-associated fatty liver disease; T_2_DM: type 2 diabetes mellitus; OR: odds ratio; CI: confidence interval; BMI: body mass index; AI: atherosclerosis index; HDL-C: high-density lipoprotein cholesterol; R-CHR: coronary heart index; AIP: atherogenic index of plasma; LCI: lipid comprehensive index; BLCI: bilirubin lipid composite index; TG: triglyceride; BAI: body adiposity index; BRI: body roundness index; LAP: lipid accumulation product; VAI: visceral fat index; WTI: waist triglyceride index; WWI: weight-adjusted waist index; CVAI: Chinese visceral adiposity index; FLI: fatty liver index; ZJU index: Zhejiang University index; TyG index: triglyceride-glucose index; FLDI: fatty liver disease index; CMI: cardiometabolic index.

**Table S2. Logistic regression analysis of assessing the relationship between 22 indices and MAFLD with adjusted age and gender**

|  | Indices | adjusted OR | 95%CI | P |
| --- | --- | --- | --- | --- |
| MAFLD |  |  |  |  |
|  | BMI | 2.184 | (1.967,2.425) | <0.05 |
|  | AI1 | 1.264 | (1.211,1.319) | <0.05 |
|  | AI2 | 1.215 | (1.168,1.265) | <0.05 |
|  | non-HDL-C | 1.179 | (1.129,1.232) | <0.05 |
|  | R-CHR | 1.264 | (1.211,1.319) | <0.05 |
|  | AIP | 1.378 | (1.320,1.438) | <0.05 |
|  | LCI | 1.559 | (1.443,1.683) | <0.05 |
|  | BLCI1 | 1.088 | (1.043,1.134) | <0.05 |
|  | BLCI2 | 1.107 | (1.061,1.155) | <0.05 |
|  | TG/HDL-C | 1.639 | (1.515,1.773) | <0.05 |
|  | BAI | 1.622 | (1.506,1.748) | <0.05 |
|  | BRI | 2.255 | (2.022,2.514) | <0.05 |
|  | LAP | 5.302 | (4.213,6.672) | <0.05 |
|  | VAI | 1.717 | (1.584,1.862) | <0.05 |
|  | WTI | 3.815 | (3.114,4.674) | <0.05 |
|  | WWI | 1.567 | (1.452,1.691) | <0.05 |
|  | FLI | 6.712 | (4.766,9.452) | <0.05 |
|  | ZJU index | 2.192 | (1.984,2.421) | <0.05 |
|  | TyG index | 1.592 | (1.492,1.700) | <0.05 |
|  | FLDI | 2.203 | (1.994,2.435) | <0.05 |
|  | CMI | 1.892 | (1.720,2.081) | <0.05 |
| Obesity  MAFLD |  |  |  |  |
|  | BMI | 1.341 | (1.262,1.425) | <0.05 |
|  | AI1 | 1.191 | (1.131,1.254) | <0.05 |
|  | AI2 | 1.146 | (1.092,1.203) | <0.05 |
|  | non-HDL-C | 1.149 | (1.086,1.215) | <0.05 |
|  | R-CHR | 1.191 | (1.131,1.254) | <0.05 |
|  | AIP | 1.261 | (1.203,1.322) | <0.05 |
|  | LCI | 1.403 | (1.285,1.532) | <0.05 |
|  | BLCI1 | 1.064 | (1.010,1.120) | <0.05 |
|  | BLCI2 | 1.074 | (1.019,1.133) | <0.05 |
|  | TG/HDL-C | 1.418 | (1.302,1.545) | <0.05 |
|  | BAI | 1.522 | (1.386,1.672) | <0.05 |
|  | BRI | 1.497 | (1.379,1.625) | <0.05 |
|  | LAP | 2.689 | (2.182,3.313) | <0.05 |
|  | VAI | 1.459 | (1.337,1.591) | <0.05 |
|  | WTI | 2.404 | (1.955,2.956) | <0.05 |
|  | WWI | 1.370 | (1.251,1.499) | <0.05 |
|  | FLI | 2.088 | (1.724,2.529) | <0.05 |
|  | ZJU index | 1.469 | (1.375,1.568) | <0.05 |
|  | TyG index | 1.355 | (1.272,1.442) | <0.05 |
|  | FLDI | 1.479 | (1.382,1.581) | <0.05 |
|  | CMI | 1.565 | (1.413,1.734) | <0.05 |
| Lean MAFLD |  |  |  |  |
|  | BMI | 2.280 | (1.583,3.283) | <0.05 |
|  | AI1 | 1.198 | (1.085,1.323) | <0.05 |
|  | AI2 | 1.184 | (1.074,1.304) | <0.05 |
|  | non-HDL-C | 1.084 | (0.998,1.176) | >0.05 |
|  | R-CHR | 1.198 | (1.085,1.323) | <0.05 |
|  | AIP | 1.516 | (1.306,1.759) | <0.05 |
|  | LCI | 1.319 | (1.157,1.505) | <0.05 |
|  | BLCI1 | 1.119 | (1.032,1.214) | <0.05 |
|  | BLCI2 | 1.127 | (1.045,1.216) | <0.05 |
|  | TG/HDL-C | 1.775 | (1.422,2.215) | <0.05 |
|  | BAI | 1.140 | (1.033,1.258) | <0.05 |
|  | BRI | 1.750 | (1.408,2.175) | <0.05 |
|  | LAP | 3.238 | (2.226,4.710) | <0.05 |
|  | VAI | 1.847 | (1.470,2.322) | <0.05 |
|  | WTI | 3.512 | (2.286,5.395) | <0.05 |
|  | WWI | 1.478 | (1.242,1.759) | <0.05 |
|  | FLI | 2.725 | (1.916,3.876) | <0.05 |
|  | ZJU index | 2.242 | (1.687,2.980) | <0.05 |
|  | TyG index | 1.869 | (1.496,2.334) | <0.05 |
|  | FLDI | 2.399 | (1.760,3.270) | <0.05 |
|  | CMI | 1.886 | (1.494,2.379) | <0.05 |
| T_2_DM MAFLD |  |  |  |  |
|  | BMI | 3.237 | (1.889,5.548) | <0.05 |
|  | AI1 | 1.153 | (1.029,1.293) | <0.05 |
|  | AI2 | 1.161 | (1.041,1.296) | <0.05 |
|  | non-HDL-C | 1.069 | (0.972,1.175) | >0.05 |
|  | R-CHR | 1.153 | (1.029,1.293) | <0.05 |
|  | AIP | 1.309 | (1.149,1.492) | <0.05 |
|  | LCI | 1.206 | (1.046,1.392) | <0.05 |
|  | BLCI1 | 1.062 | (0.942,1.198) | >0.05 |
|  | BLCI2 | 1.088 | (0.969,1.222) | >0.05 |
|  | TG/HDL-C | 1.276 | (1.090,1.493) | <0.05 |
|  | BAI | 1.827 | (1.337,2.496) | <0.05 |
|  | BRI | 4.641 | (2.228,9.667) | <0.05 |
|  | LAP | 5.017 | (2.399,10.489) | <0.05 |
|  | VAI | 1.552 | (1.216,1.981) | <0.05 |
|  | WTI | 2.798 | (1.551,5.050) | <0.05 |
|  | WWI | 1.544 | (1.244,1.915) | <0.05 |
|  | FLI | 14.725 | (3.712,58.420) | <0.05 |
|  | ZJU index | 1.910 | (1.441,2.531) | <0.05 |
|  | TyG index | 1.408 | (1.174,1.689) | <0.05 |
|  | FLDI | 2.543 | (1.700,3.803) | <0.05 |
|  | CMI | 1.438 | (1.179,1.755) | <0.05 |

The correlations between the indexes and MAFLD groups were analyzed by multivariate logistic regression.

MAFLD: metabolic dysfunction-associated fatty liver disease; T_2_DM: type 2 diabetes mellitus; OR: odds ratio; adjusted OR: the values of ORs adjusted for gender and age (for age of LAP, VAI, and ZJU index); CI: confidence interval; BMI: body mass index; AI: atherosclerosis index; HDL-C: high-density lipoprotein cholesterol; R-CHR: coronary heart index; AIP: atherogenic index of plasma; LCI: lipid comprehensive index; BLCI: bilirubin lipid composite index; TG: triglyceride; BAI: body adiposity index; BRI: body roundness index; LAP: lipid accumulation product; VAI: visceral fat index; WTI: waist triglyceride index; WWI: weight-adjusted waist index; FLI: fatty liver index; ZJU index: Zhejiang University index; TyG index: triglyceride-glucose index; FLDI: fatty liver disease index; CMI: cardiometabolic index.

**Table S3. Prevalence of MAFLD and its subgroups in FLI quartile groups [n(%)]**

|  | FLI | | | | χ^2^ | *P* | *Cramer's* V |
| --- | --- | --- | --- | --- | --- | --- | --- |
|  | F1 (n=265) | F2 (n=264) | F3 (n=264) | F4(n=265) |  |  |  |
| MAFLD | 27 (10.19) | 137 (51.89)*^a^* | 213 (80.68)*^ab^* | 244 (92.08)*^abc^* | 436.655 | <0.01 | 0.642 |
| non-MAFLD | 238 (89.81) | 127 (48.11)*^a^* | 51 (19.32)*^ab^* | 21 (7.92)*^abc^* |  |  |  |
| OR |  | 9.509*^*^* | 36.815*^*^* | 102.420*^*^* |  |  |  |
| adjusted OR |  | 12.830*^*^* | 49.884*^*^* | 149.589*^*^* |  |  |  |
| Obesity MAFLD | 16 (6.04) | 111 (42.05)*^a^* | 185 (70.08)*^ab^* | 152 (57.36)*^abc^* | 183.774 | <0.01 | 0.509 |
| Obesity non-MAFLD | 81 (30.57) | 100 (37.88)*^a^* | 44 (16.67)*^ab^* | 19 (7.17)*^abc^* |  |  |  |
| OR |  | 5.619*^*^* | 21.286*^*^* | 40.500*^*^* |  |  |  |
| adjusted OR |  | 6.616*^*^* | 24.854*^*^* | 53.045*^*^* |  |  |  |
| Lean MAFLD | 9 (3.40) | 21 (7.95)*^a^* | 6 (2.27)*^a^* | 0 | 73.659 | <0.01 | 0.626 |
| Lean non-MAFLD | 137 (51.70) | 14 (5.30)*^a^* | 1 (0.38)*^a^* | 0 |  |  |  |
| OR |  | 22.833*^*^* | 91.333*^*^* | - |  |  |  |
| adjusted OR |  | 26.790*^*^* | 61.500*^*^* | - |  |  |  |
| T_2_DM MAFLD | 2 (0.75) | 5 (1.89) | 22 (8.33)*^ab^* | 92 (34.72)*^abc^* | 97.985 | <0.01 | 0.778 |
| T_2_DM non-MAFLD | 20 (7.55) | 13 (4.92) | 6 (2.27)*^ab^* | 2 (0.75)*^abc^* |  |  |  |
| OR |  | 3.846 | 36.667*^*^* | 460.000*^*^* |  |  |  |
| adjusted OR |  | 4.962 | 70.418*^*^* | 321.323*^*^* |  |  |  |

The correlations between FLI and MAFLD groups in quartile groups were analyzed by univariate and multivariate logistic regression. Data were expressed as number (percentage) and analyzed by chi-square test for categorical variables.

Notes: compared with F1, *^*^P*＜0.05; compared with F1, *^a^P*＜0.05; compared with F2, *^b^P*＜0.05; compared with F3, *^c^P*＜0.05.

MAFLD: metabolic dysfunction-associated fatty liver disease; T_2_DM: type 2 diabetes mellitus; OR: odds ratio; adjusted OR: the value of ORs after adjusting for gender and age; FLI: fatty liver index.

**Table S4. Prevalence of MAFLD and its subgroups in LAP quartile groups [n(%)]**

|  | LAP | | | | χ^2^ | *P* | Cramer's V |
| --- | --- | --- | --- | --- | --- | --- | --- |
|  | L1 (n=265) | L2 (n=264) | L3 (n=264) | L4(n=265) |  |  |  |
| MAFLD | 26 (9.81) | 142 (53.79)*^a^* | 207 (78.41)*^ab^* | 246 (92.83)*^abc^* | 433.507 | <0.01 | 0.640 |
| non-MAFLD | 239 (90.19) | 122 (46.21)*^a^* | 57 (21.59)*^ab^* | 19 (7.17)*^abc^* |  |  |  |
| OR |  | 10.699*^*^* | 33.383*^*^* | 119.016*^*^* |  |  |  |
| adjusted OR |  | 10.678*^*^* | 33.184*^*^* | 118.640*^*^* |  |  |  |
| Obesity MAFLD | 21 (7.92) | 125 (47.35)*^a^* | 165 (62.50)*^ab^* | 153 (57.74)*^abc^* | 175.858 | <0.01 | 0.498 |
| Obesity non-MAFLD | 89 (33.58) | 95 (35.98)*^a^* | 44 (16.67)*^ab^* | 16 (6.04)*^abc^* |  |  |  |
| OR |  | 5.576*^*^* | 15.893*^*^* | 40.527*^*^* |  |  |  |
| adjusted OR |  | 5.521*^*^* | 15.461*^*^* | 39.894*^*^* |  |  |  |
| Lean MAFLD | 3 (1.13) | 11 (4.17)*^a^* | 15 (5.68)*^ab^* | 7 (2.64)*^ab^* | 97.285 | <0.01 | 0.719 |
| Lean non-MAFLD | 128 (48.30) | 18 (6.82)*^a^* | 6 (2.27)*^ab^* | 0*^ab^* |  |  |  |
| OR |  | 26.074*^*^* | 106.667*^*^* | - |  |  |  |
| adjusted OR |  | 24.695*^*^* | 95.416*^*^* | - |  |  |  |
| T_2_DM MAFLD | 2 (0.75) | 6 (2.27)*^a^* | 27 (10.23)*^ab^* | 86 (32.45)*^abc^* | 88.516 | <0.01 | 0.739 |
| T_2_DM non-MAFLD | 22 (8.30) | 9 (3.41)*^a^* | 7 (2.65)*^ab^* | 3 (1.13)*^abc^* |  |  |  |
| OR |  | 7.333*^*^* | 42.429*^*^* | 315.333*^*^* |  |  |  |
| adjusted OR |  | 5.051 | 36.671*^*^* | 95.514*^*^* |  |  |  |

The correlations between LAP and MAFLD groups in quartile groups were analyzed by univariate and multivariate logistic regression. Data were expressed as number (percentage) and analyzed by chi-square test for categorical variables.

Notes: compared with L1, *^*^P*＜0.05; compared with L1, *^a^P*＜0.05; compared with L2, *^b^P*＜0.05; compared with L3, *^c^P*＜0.05.

MAFLD: metabolic dysfunction-associated fatty liver disease; T_2_DM: type 2 diabetes mellitus; OR: odds ratio; adjusted OR: the values of ORs after adjusting for age; LAP: lipid accumulation product.

**Table S5. Prevalence of MAFLD and its subgroups in WTI quartile groups [n(%)]**

|  | WTI | | | | χ^2^ | *P* | Cramer's V |
| --- | --- | --- | --- | --- | --- | --- | --- |
|  | W1 (n=265) | W2 (n=264) | W3 (n=264) | W4(n=265) |  |  |  |
| MAFLD | 44 (16.60) | 141 (53.41)*^a^* | 196 (74.24)*^ab^* | 240 (90.57)*^abc^* | 334.048 | <0.01 | 0.562 |
| non-MAFLD | 221 (83.40) | 123 (46.59)*^a^* | 68 (25.76)*^ab^* | 25 (9.43)*^abc^* |  |  |  |
| OR |  | 5.758*^*^* | 14.477*^*^* | 48.218*^*^* |  |  |  |
| adjusted OR |  | 6.315*^*^* | 16.248*^*^* | 55.893*^*^* |  |  |  |
| Obesity MAFLD | 38 (14.34) | 122 (46.21)*^a^* | 142 (53.79)*^ab^* | 162 (61.13)*^abc^* | 128.948 | <0.01 | 0.427 |
| Obesity non-MAFLD | 91 (34.34) | 84 (31.82)*^a^* | 49 (18.56)*^ab^* | 20 (7.55)*^abc^* |  |  |  |
| OR |  | 3.478*^*^* | 6.940*^*^* | 19.397*^*^* |  |  |  |
| adjusted OR |  | 3.557 | 7.212*^*^* | 21.159*^*^* |  |  |  |
| Lean MAFLD | 3 (1.13) | 8 (3.03)*^a^* | 19 (7.20)*^ab^* | 6 (2.26)*^ab^* | 78.098 | <0.01 | 0.645 |
| Lean non-MAFLD | 111 (41.89) | 29 (10.98)*^a^* | 11 (4.17)*^ab^* | 1 (0.38)*^ab^* |  |  |  |
| OR |  | 10.207*^*^* | 63.909*^*^* | 222.000*^*^* |  |  |  |
| adjusted OR |  | 12.980*^*^* | 82.272*^*^* | 221.349*^*^* |  |  |  |
| T_2_DM MAFLD | 3 (1.13) | 11 (4.17)*^a^* | 35 (13.26)*^ab^* | 72 (27.17)*^abc^* | 66.091 | <0.01 | 0.639 |
| T_2_DM non-MAFLD | 19 (7.17) | 10 (3.79)*^a^* | 8 (3.03)*^ab^* | 4 (1.51)*^abc^* |  |  |  |
| OR |  | 6.967*^*^* | 27.708*^*^* | 114.000*^*^* |  |  |  |
| adjusted OR |  | 5.564 | 38.402*^*^* | 53.650*^*^* |  |  |  |

The correlations between WTI and MAFLD groups in quartile groups were analyzed by univariate and multivariate logistic regression. Data were expressed as number (percentage) and analyzed by chi-square test for categorical variables.

Notes: compared with W1, *^*^P*＜0.05; compared with W1, *^a^P*＜0.05; compared with W2, *^b^P*＜0.05; compared with W3, *^c^P*＜0.05.

MAFLD: metabolic dysfunction-associated fatty liver disease; T_2_DM: type 2 diabetes mellitus; OR: odds ratio; adjusted OR: the value of ORs after adjusting for gender and age; WTI: waist triglyceride index.

**Table S6. ROC analysis of 22 indices in predicting the risk of MAFLD and its subgroups**

|  | Indices | AUC | 95%CI | Sensitivity  (%) | Specificity  (%) | Youden index | Cutoff points |
| --- | --- | --- | --- | --- | --- | --- | --- |
| MAFLD |  |  |  |  |  |  |  |
|  | BMI | 0.836 | (0.812,0.858) | 84.38 | 65.90 | 0.5028 | 25.39 |
|  | AI1 | 0.728 | (0.700,0.755) | 65.06 | 70.02 | 0.3508 | 2.84 |
|  | AI2 | 0.699 | (0.671,0.727) | 68.44 | 62.93 | 0.3137 | 2.18 |
|  | non-HDL-C | 0.659 | (0.630,0.688) | 64.57 | 60.41 | 0.2499 | 3.35 |
|  | R-CHR | 0.728 | (0.700,0.755) | 65.06 | 70.02 | 0.3508 | 3.84 |
|  | AIP | 0.810 | (0.785,0.833) | 79.87 | 70.02 | 0.4989 | 0.03 |
|  | LCI | 0.787 | (0.761,0.811) | 71.82 | 72.31 | 0.4413 | 15.46 |
|  | BLCI1 | 0.562 | (0.531,0.592) | 35.43 | 75.51 | 0.1094 | 0.41 |
|  | BLCI2 | 0.579 | (0.548,0.609) | 66.18 | 47.14 | 0.1332 | 0.17 |
|  | TG/HDL-C | 0.810 | (0.785,0.833) | 79.87 | 70.02 | 0.4989 | 1.08 |
|  | BAI | 0.747 | (0.720,0.773) | 73.59 | 62.70 | 0.3629 | 27.24 |
|  | BRI | 0.839 | (0.815,0.860) | 85.83 | 64.30 | 0.5013 | 3.79 |
|  | LAP | 0.873 | (0.851,0.892) | 86.15 | 72.54 | 0.5869 | 36.51 |
|  | VAI | 0.825 | (0.801,0.847) | 81.96 | 70.25 | 0.5222 | 1.57 |
|  | WTI | 0.835 | (0.811,0.857) | 84.70 | 66.82 | 0.5152 | 113.96 |
|  | WWI | 0.724 | (0.695,0.750) | 83.57 | 49.89 | 0.3346 | 10.20 |
|  | CVAI | 0.843 | (0.820,0.864) | 84.38 | 69.11 | 0.5349 | 100.11 |
|  | FLI | 0.879 | (0.857,0.898) | 88.57 | 72.31 | 0.6088 | 0.91 |
|  | ZJU index | 0.876 | (0.854,0.895) | 82.13 | 75.74 | 0.5787 | 37.74 |
|  | TyG index | 0.807 | (0.782,0.831) | 71.34 | 75.51 | 0.4685 | 1.37 |
|  | FLDI | 0.873 | (0.852,0.893) | 76.17 | 81.01 | 0.5717 | 32.74 |
|  | CMI | 0.839 | (0.815,0.860) | 80.03 | 74.60 | 0.5463 | 0.61 |
| Obesity  MAFLD |  |  |  |  |  |  |  |
|  | BMI | 0.748 | (0.715,0.780) | 62.50 | 72.54 | 0.3504 | 27.14 |
|  | AI1 | 0.673 | (0.637,0.707) | 78.02 | 48.36 | 0.2638 | 2.50 |
|  | AI2 | 0.645 | (0.608,0.680) | 48.06 | 75.41 | 0.2347 | 2.59 |
|  | non-HDL-C | 0.628 | (0.591,0.664) | 63.36 | 57.38 | 0.2074 | 3.34 |
|  | R-CHR | 0.673 | (0.637,0.707) | 78.02 | 48.36 | 0.2638 | 3.50 |
|  | AIP | 0.740 | (0.707,0.772) | 77.16 | 61.89 | 0.3904 | 0.03 |
|  | LCI | 0.726 | (0.692,0.759) | 59.27 | 76.23 | 0.3550 | 19.16 |
|  | BLCI1 | 0.554 | (0.517,0.591) | 45.26 | 64.75 | 0.1001 | 0.37 |
|  | BLCI2 | 0.560 | (0.522,0.597) | 67.03 | 43.85 | 0.1088 | 0.17 |
|  | TG/HDL-C | 0.740 | (0.707,0.772) | 77.16 | 61.89 | 0.3904 | 1.08 |
|  | BAI | 0.694 | (0.659,0.728) | 73.49 | 53.28 | 0.2677 | 27.06 |
|  | BRI | 0.744 | (0.711,0.776) | 71.12 | 63.93 | 0.3506 | 4.12 |
|  | LAP | 0.796 | (0.765,0.826) | 64.01 | 82.79 | 0.4680 | 50.70 |
|  | VAI | 0.754 | (0.720,0.785) | 78.88 | 62.30 | 0.4117 | 1.57 |
|  | WTI | 0.762 | (0.728,0.792) | 63.36 | 75.82 | 0.3918 | 149.38 |
|  | WWI | 0.658 | (0.622,0.693) | 80.39 | 43.44 | 0.2383 | 10.20 |
|  | CVAI | 0.769 | (0.737,0.800) | 76.51 | 67.62 | 0.4413 | 109.37 |
|  | FLI | 0.806 | (0.774,0.834) | 75.86 | 72.13 | 0.4799 | 1.49 |
|  | ZJU index | 0.816 | (0.786,0.844) | 71.12 | 80.33 | 0.5145 | 39.14 |
|  | TyG index | 0.745 | (0.711,0.777) | 66.38 | 70.49 | 0.3687 | 1.37 |
|  | FLDI | 0.810 | (0.779,0.838) | 73.92 | 73.77 | 0.4769 | 33.2 |
|  | CMI | 0.763 | (0.730,0.794) | 76.94 | 65.16 | 0.4210 | 0.61 |
| Lean MAFLD |  |  |  |  |  |  |  |
|  | BMI | 0.890 | (0.836,0.931) | 86.11 | 83.55 | 0.6966 | 22.48 |
|  | AI1 | 0.735 | (0.666,0.796) | 86.11 | 51.32 | 0.3743 | 2.02 |
|  | AI2 | 0.700 | (0.629,0.764) | 55.56 | 77.63 | 0.3319 | 2.16 |
|  | non-HDL-C | 0.676 | (0.604,0.742) | 75.00 | 58.55 | 0.3355 | 3.09 |
|  | R-CHR | 0.735 | (0.666,0.796) | 86.11 | 51.32 | 0.3743 | 3.02 |
|  | AIP | 0.880 | (0.824,0.922) | 80.56 | 80.92 | 0.6148 | -0.01 |
|  | LCI | 0.853 | (0.794,0.900) | 94.44 | 66.45 | 0.6089 | 9.21 |
|  | BLCI1 | 0.683 | (0.611,0.749) | 86.11 | 50.00 | 0.3611 | 0.32 |
|  | BLCI2 | 0.706 | (0.636,0.770) | 72.22 | 65.79 | 0.3801 | 0.20 |
|  | TG/HDL-C | 0.880 | (0.824,0.922) | 80.56 | 80.92 | 0.6148 | 0.98 |
|  | BAI | 0.645 | (0.572,0.713) | 83.33 | 41.45 | 0.2478 | 24.40 |
|  | BRI | 0.886 | (0.832,0.928) | 86.11 | 83.55 | 0.6966 | 3.25 |
|  | LAP | 0.951 | (0.910,0.977) | 91.67 | 88.82 | 0.8048 | 28.12 |
|  | VAI | 0.923 | (0.876,0.957) | 91.67 | 81.58 | 0.7325 | 1.50 |
|  | WTI | 0.920 | (0.872,0.954) | 80.56 | 89.47 | 0.7003 | 120.12 |
|  | WWI | 0.829 | (0.767, 0.880) | 86.11 | 71.71 | 0.5782 | 10.27 |
|  | CVAI | 0.911 | (0.861,0.948) | 88.89 | 86.18 | 0.7507 | 71.05 |
|  | FLI | 0.940 | (0.896,0.969) | 97.22 | 81.58 | 0.7880 | 0.28 |
|  | ZJU index | 0.952 | (0.912,0.978) | 88.89 | 89.47 | 0.7836 | 33.71 |
|  | TyG index | 0.909 | (0.858,0.946) | 100.00 | 68.42 | 0.6842 | 0.99 |
|  | FLDI | 0.943 | (0.900,0.971) | 88.89 | 88.82 | 0.7770 | 27.63 |
|  | CMI | 0.912 | (0.863,0.949) | 80.56 | 88.16 | 0.6871 | 0.54 |
| T_2_DM MAFLD |  |  |  |  |  |  |  |
|  | BMI | 0.953 | (0.909,0.980) | 96.69 | 78.05 | 0.7474 | 25.99 |
|  | AI1 | 0.756 | (0.682,0.820) | 79.34 | 65.85 | 0.4519 | 2.88 |
|  | AI2 | 0.747 | (0.673,0.812) | 83.47 | 56.10 | 0.3957 | 1.91 |
|  | non-HDL-C | 0.635 | (0.556,0.709) | 70.25 | 58.54 | 0.2878 | 3.46 |
|  | R-CHR | 0.756 | (0.682,0.820) | 79.34 | 65.85 | 0.4519 | 3.88 |
|  | AIP | 0.852 | (0.788,0.903) | 80.99 | 78.05 | 0.5904 | 0.14 |
|  | LCI | 0.835 | (0.769,0.889) | 82.64 | 73.17 | 0.5582 | 15.96 |
|  | BLCI1 | 0.548 | (0.468,0.626) | 23.97 | 90.24 | 0.1421 | 0.47 |
|  | BLCI2 | 0.581 | (0.501,0.658) | 68.60 | 51.22 | 0.1981 | 0.15 |
|  | TG/HDL-C | 0.852 | (0.788,0.903) | 80.99 | 78.05 | 0.5904 | 1.38 |
|  | BAI | 0.874 | (0.813,0.921) | 83.47 | 80.49 | 0.6396 | 27.79 |
|  | BRI | 0.963 | (0.921,0.986) | 81.82 | 100.00 | 0.8182 | 4.97 |
|  | LAP | 0.934 | (0.885,0.967) | 90.08 | 87.80 | 0.7789 | 56.16 |
|  | VAI | 0.890 | (0.831,0.934) | 87.60 | 82.93 | 0.7053 | 2.07 |
|  | WTI | 0.877 | (0.816,0.923) | 84.30 | 82.93 | 0.6722 | 159.08 |
|  | WWI | 0.808 | (0.739,0.866) | 83.47 | 70.73 | 0.5420 | 10.62 |
|  | CVAI | 0.894 | (0.836,0.936) | 70.25 | 97.56 | 0.6781 | 151.07 |
|  | FLI | 0.958 | (0.915,0.983) | 90.91 | 87.80 | 0.7871 | 2.07 |
|  | ZJU index | 0.945 | (0.897,0.974) | 82.64 | 92.68 | 0.7533 | 43.57 |
|  | TyG index | 0.809 | (0.740,0.866) | 89.26 | 60.98 | 0.5023 | 1.48 |
|  | FLDI | 0.951 | (0.906,0.979) | 84.30 | 95.12 | 0.7942 | 36.54 |
|  | CMI | 0.891 | (0.832,0.934) | 92.56 | 75.61 | 0.6817 | 0.70 |

The predictive abilities of indicators were analyzed by ROC.

MAFLD: metabolic dysfunction-associated fatty liver disease; T_2_DM: type 2 diabetes mellitus; ROC: receiver operating characteristic; AUC: area under the curve; CI: confidence interval; BMI: body mass index; AI: atherosclerosis index; HDL-C: high-density lipoprotein cholesterol; R-CHR: coronary heart index; AIP: atherogenic index of plasma; LCI: lipid comprehensive index; BLCI: bilirubin lipid composite index; TG: triglyceride; BAI: body adiposity index; BRI: body roundness index; LAP: lipid accumulation product; VAI: visceral fat index; WTI: waist triglyceride index; WWI: weight-adjusted waist index; CVAI: Chinese visceral adiposity index; FLI: fatty liver index; ZJU index: Zhejiang University index; TyG index: triglyceride-glucose index; FLDI: fatty liver disease index; CMI: cardiometabolic index.
